# Effective methane production from the Japanese weed Gyougi-shiba (*Cynodon dactylon*) is accomplished by colocalization of microbial communities that assimilate water-soluble and -insoluble fractions

**DOI:** 10.1093/femsle/fnab015

**Published:** 2021-02-15

**Authors:** Shuhei Matsuda, Takashi Ohtsuki

**Affiliations:** Graduate School of Medicine, Engineering and Agricultural Sciences, University of Yamanashi, Kofu 400-8510, Yamanashi, Japan; Graduate School of Medicine, Engineering and Agricultural Sciences, University of Yamanashi, Kofu 400-8510, Yamanashi, Japan

**Keywords:** methane production, microbial community, weed, lignocellulose, nanopore sequencing, soluble and insoluble fractions

## Abstract

Weed, an abundant biomass, is considered unsuitable as a raw material for methane production. There are few reports on the anaerobic digestion of weeds without the addition of other organic wastes. To solve this problem, a methane-producing microbial community with weed as a sole feedstock was established. This study mainly focused on the degree of contribution between water-soluble and -insoluble fractions of the weed to methane production; thus, methane production from both fractions was tested separately. Methane production after 80-day batch cultures with whole weed, water-soluble and water-insoluble fractions was 184.5, 96.8 and 26.5 NmL g^−1^ dry matter (DM), respectively. The results of 16S rRNA gene amplicon sequence analysis revealed that *Proteiniphilum saccharofermentans* and several *Methanobacterium* species commonly dominated all cultures, whereas the population dynamics of minor species differed in every culture. Moreover, the remixed culture of microbial communities adapted to water-soluble and -insoluble fractions recovered methane production (252.4 NmL g^−1^ DM). Based on these results, it can be strongly inferred that colocalizing the minor species in water-soluble and -insoluble fractions is important for effective methane production.

## Teaser

Methane production from the Japanese weed Gyougi-shiba, a sole feedstock, is required for the coexistence of water-soluble and -insoluble fractions of the weed for full-performance production.

## INTRODUCTION

With the depletion of fossil fuels and global warming becoming a nationwide concern, anaerobic digestion is being considered as a technology for using biomass (Kaparaju *et al*. 2009). In anaerobic digestion, eubacteria hydrolyze the substrate and generate acid, and then methanogens produce methane from hydrogen, carbon dioxide and acetate (Dahiya *et al*. 2018).

Anaerobic digestion is an excellent technology that can produce methane from various biomass feedstock, but it is unsuitable for digesting lignocellulosic biomass, including weed. When lignocellulose is used as a substrate, the hydrolysis step is the rate-limiting step because few bacteria have cellulose degradation capacity and lignin hinders cellulose assimilation (Himmel *et al*. 2007; Kumar, Singh and Singh 2008). Methane yield in anaerobic digestion using lignocellulosic biomass mainly depends on the ratio of polysaccharides to lignin in the feedstock (Klimiuk *et al*. 2010). Therefore, various pretreatment methods, such as chemical, physical and biological processes, have been studied for improving cellulose hydrolysis efficiency and lignin removal (Hendriks and Zeeman 2009; Rodriguez *et al*. 2017). In lignocellulose pretreatment, the loss of substrate components during pretreatment was recognized. For example, Ashraf *et al*. (2017) reported the dissolution of glucose and xylose in the liquid fraction of Bermuda grass after hydrothermal treatment. Moreover, according to Kothari *et al*. (2018), hydrothermal treatment of switchgrass greatly enhanced the solubilization of glucan by *Clostridium thermocellum*. Additionally, Merali *et al*. (2013) reported that pretreatment of wheat straw using the hydrothermal technique induced hemicellulose elution. However, there are few studies on the effect of the components eluted from the substrate in the pretreatment process on the methanogenic microbial community.

Weed is a globally abundant biomass and therefore a potential source of bioenergy production. However, anaerobic digestion as a major biological waste treatment method has been considered unfavorable for converting weed as a sole feedstock into methane. In our previous study, we succeeded in establishing microbial communities that can produce 322 mL g^-1^ dry matter (DM) of methane from weed as a sole feedstock (Matsuda and Ohtsuki 2016). In the experiments, we also observed the elution of water-soluble components from weed into the medium by autoclaving. The degree of contribution of soluble components toward total methane production by the methanogenic microbial community was remarkable. Concurrently, elucidating the colocalized community structure assimilating the insoluble residue will be key for further increases in methane production.

In this study, changes in methane production, volatile fatty acid (VFA) concentration and the microbial community population were compared among batch cultures with whole weed (WW), water-soluble fraction (WSF) and water-insoluble fraction (WIF) of weed. Of particular interest, the population changes of several minor species in the microbial communities differed between the cultures with WSF and WIF, suggesting colocalization of the species in the culture with WW. Therefore, an experiment with a re-mixture of communities cultured with WSF and WIF was additionally carried out and is discussed.

## MATERIALS AND METHODS

### Feedstock weed

We used the blade, sheath and culm parts of a Bermuda grass (named ‘Gyougi-shiba’ in Japanese, taxonomically classified as *Cynodon dactylon* and having a C/N ratio of ~200, which is unlike general Bermuda grass, as it is known in Europe and the USA; hereinafter called ‘weed’), which was collected at the University of Yamanashi, dried at 80°C overnight and stored at 25°C. The weed was powdered by a mill (Model WB-1, Osaka Chemicals, Osaka, Japan), passed through a 100-μm mesh stainless steel sieve and completely dried at 105°C before use.

### Acclimation and maintenance of the inoculum

We have already established a method to obtain the microbial communities that can produce methane from weeds as a sole feedstock (Matsuda and Ohtsuki 2016). To produce methane from weeds in this study, we reacclimated the microbial community from the bottom mud of a water reservoir pond at the University of Yamanashi. Briefly, 95 mL of 0.1 M potassium sodium phosphate buffer (K_2_HPO_4_-NaH_2_PO_4_•2H_2_O, pH 7.2) containing 1 g weed powder in a 175 mL glass bottle sealed with a butyl rubber stopper and a plastic cap was autoclaved at 121°C for 20 min and used as the medium. Five milliliters of bottom mud collected from the water reservoir pond was inoculated and the head space was purged with nitrogen gas for 3 min and statically cultured at 35°C. Every 3 mo, 5 mL of culture suspension was transferred to the same fresh medium and the subculture was repeated three more times for acclimating and stabilizing the microbial community.

### Batch culture with whole and fractionated weed

To investigate the participation of microbial community members in the digestion of and methane production from weeds, we conducted batch culture experiments with WW, WSF and WIF. For the preparation of WSF and WIF, weed powder (20 g L^−1^) was added to deionized water and autoclaved at 121°C for 20 min. After cooling, the suspension was filtered under vacuum using a glass-fiber filter (GA-100, retaining particle size: >1 µm, 55 mm diameter; ADVANTEC, Tokyo, Japan) and the filtrate was used as WSF. The solid residue collected on the glass-fiber filter was completely dried and used as WIF.

Table 1 shows the composition of the media used in the batch experiments. The components without inocula were mixed in 30-mL glass vials sealed with butyl rubber stoppers and plastic caps and autoclaved. Then, in each of the autoclaved substrate-containing vials, 1 ml of the inoculum (pH 6.9) was added, followed by purging for 1 min with nitrogen gas and static culture at 35°C for 80 days.

**Table 1.** Medium composition of batch experiments.

Substrate	Cell suspension (mL)	Sterilized water (mL)	1 M PB (mL)	WW (g)	WSF (mL)	WIF (g)	Total volume (mL)
WW	1	17	2	0.2	–	–	20
Control	1	17	2	–	–	–	20
WSF	1	7	2	–	10	–	20
WIF	1	17	2	–	–	0.1	20

WW—whole weed; WSF—water-soluble fraction; WIF—water-insoluble fraction; PB—potassium sodium phosphate buffer; –, not added.

Ten milliliters of WSF contain soluble components extracted from 0.1 g-equivalent of WW.

Headspace gas and culture suspension in the vials were periodically collected for chemical and sequencing analysis. Every 10 days, the normalized volume of positive pressure gas in the vials was measured and collected using needles and syringes, after which the caps of the vials were opened and 1 mL of mixed culture suspensions were sampled. After sampling, the vials were sealed, re-purged with nitrogen gas and the culture continued.

### Analytical methods

To determine the extracted components of weed during autoclaving, we compared the components between WW and WIF. Contents of lipid, holocellulose and α-cellulose were determined using the method described by Yokoyama, Kadla and Chang (2002). The hemicellulose contents were calculated as differences between holocellulose and α-cellulose. Klason lignin contents were determined according to the description of Browning (1967).

For the determination of ash and volatile solid contents, completely dried powder of WW was placed in a crucible and ashed in a muffle furnace set at 550°C for 2 h. After ashing, the remaining components in the crucible were weighed as ash and the weight decreased by ashing was considered to be volatile solids (APHA 2005).

Methane contents in the collected gas samples were determined by gas chromatography (Model GC2014, Shimadzu, Kyoto, Japan) equipped with a Molecular Sieve 5A 60–80 column (3 mm ID × 3 m, GL Science, Tokyo, Japan) and a thermal conductivity detector. The column temperature was maintained at 40°C for 15 min and thereafter the temperature was brought to 200°C by increasing at 20°C min^-1^ then finally maintained for 20 min. The temperature of the injector and detector was 200°C. Nitrogen was used as the carrier gas (30 mL min^−1^).

The sampled culture suspensions were centrifuged at 11 000 × *g* for 7 min and the pellets were stored at −20°C for DNA analysis. The pH of the supernatants was measured using a portable meter (LAQUAtwin B-212, Horiba, Kyoto, Japan). The concentration of VFA in supernatants was determined using gas chromatography (Model GC2014) equipped with an HP-Innowax column (0.53 mm ID × 15 m, 1.0 μm thick, Agilent Technologies, CA, USA) and a flame-ionized detector. The column temperature was maintained at 100°C for 34 min and thereafter the temperature was brought to 220°C by increasing at 10°C min^−1^ then finally maintained for 25 min. The temperatures of the injector and detector were 250°C and 300°C, respectively. Helium was used as the carrier gas (3.59 mL min^−1^, 10:1 of split ratio).

### Remixed culture of subcultures with WSF and WIF

Five milliliters of culture suspension (pH 6.9) from the fifth subculture with WW was inoculated into 95 mL of the medium comprising 0.1 M potassium sodium phosphate buffer (pH 7.2) containing 10 mL WSF or 0.5 g WIF. After 3 mo, each suspension of the culture with WSF or WIF was transferred to the same fresh medium and subcultured for a further 3 mo. Subsequently, 2.5 mL of each culture suspension (pH: 7.2 of WSF and 6.8 of WIF) from the second subcultures with WSF and WIF was mixed and inoculated into the fresh medium with WW (abbreviated as ‘Mix’). All cultures were performed anaerobically under purged nitrogen gas and statically at 35°C.

### 16S rRNA gene amplicon sequence analysis using a nanopore sequencer

Genomic DNA was extracted from the pellets harvested from the culture according to a previously described method (Zhu, Qu and Zhu 1993). Ten nanograms of extracted DNA were amplified by PCR with the Tks Gflex DNA polymerase (Takara Bio, Shiga, Japan). The primer sets Pro341F (5′-CCTACGGGNBGCASCAG-3′) and Pro805R (5′-GACTACNVGGGTATCTAATCC-3′) were used (Takahashi *et al*. 2014). PCR amplification was performed by an initial denaturation at 98°C for 2 min, followed by 35 cycles of denaturation at 98°C for 10 s, annealing at 65°C for 15 s (for the first 11 cycles, the temperature was decreased by 1°C for each cycle to reach 55°C and the remaining 24 cycles were performed at 55°C) and elongation at 68°C for 30 s. Purification and barcoding of PCR products were performed using PCR Barcoding Kit (SQK-PBK004, Oxford Nanopore Technologies, Oxford, UK). AxyPrep MAG Clean-Up kit (Corning, NY, USA) was used for all purification steps of the PCR products. Qubit dsDNA HS Assay Kit (Thermo Fisher Scientific, MA, USA) was used to determine the concentration of genomic DNA and PCR products. Twelve barcoded PCR products were mixed at 8 fmoles each and loaded on a flow cell (FLO-MIN106 R9, Oxford Nanopore Technologies); sequencing was performed using MinION Mk1B apparatus. Sequenced data collecting, base calling and quality checking were obtained with MinION software (release 18.12.6, Oxford Nanopore Technologies) to obtain fastq files. The FASTQ BARCODING workflow (v. 3.10.2, Metrichor, Oxford, UK) was used to remove barcode sequences and the FASTQ WIMP workflow (v. 3.2.1, Metrichor) was used for taxonomic assignment of the cleaned reads. The WIMP workflow can indicate the population ratio in microbial communities at the species level based on a novel classification engine ‘Centrifuge’ (Kim *et al*. 2016) and we illustrated ‘growth curve-like’ using the population data to compare structures and changes among bacterial communities in the examined cultures.

### Statistical analysis

Significant differences in methane production and VFA concentrations were tested using the TukeyHSD function in the RStudio software (v. 1.1.383, RStudio, MA, USA). Analysis of differences in microbial community structures was performed using Metagenome@KIN software (v. 2.0.0, World Fusion, Tokyo, Japan), which was based on the R package (v. 3.1.0, The R Foundation, Vienna, Austria). The fastq sequences with ≥97% similarity were assigned to the same operational taxonomic units (OTUs). Representative sequences for each OTU were used for taxonomic annotation with BLAST. The numbers of OTUs were log-transformed when necessary to normalize the distribution and to achieve homogeneity of variance. To illustrate the analysis results, the ClustVis web tool (https://biit.cs.ut.ee/clustvis/) was used.

## RESULTS AND DISCUSSION

### Determination of amounts of weed components dissolved in the medium

Table 2 shows the components determined in WW and WIF. The contents of volatile solids and ash in WW were 0.905 ± 0.005 g and 0.095 ± 0.005 g per gram of dry WW, respectively. Hemicellulose, α-cellulose and other components (mainly comprising amino acids and proteins) were decreased in WIF by autoclaving, indicating that ~50% (w w^−1^) of the total weed components were dissolved into the medium.

**Table 2.** Components of Gyogi-shiba in solid form before and after autoclaving.

	Content ± standard deviations	
Component	Before (g g^-1^ DM)	After (g)*
Lipid	0.0125 ± 0.010	0.017 ± 0.008
Hemicellulose	0.317 ± 0.039	0.232 ± 0.009
α-cellulose	0.127 ± 0.042	0.059 ± 0.005
Lignin	0.163 ± 0.009	0.200 ± 0.040
Others	0.381 ± 0.050	0.004 ± 0.041

Component analysis was performed in triplicate.

* Weights are indicated as contents in water-insoluble residue obtained from 1 g of Gyogi-shiba powder.

Based on these results, WSF contained a considerable amount of nutrient source and we decided to investigate the dynamics of microbial communities in the culture with WSF and WIF separately.

### Comparison of changes in cumulative methane production, pH and VFA concentrations among cultures with WW, WSF and WIF

**Figure 1.**
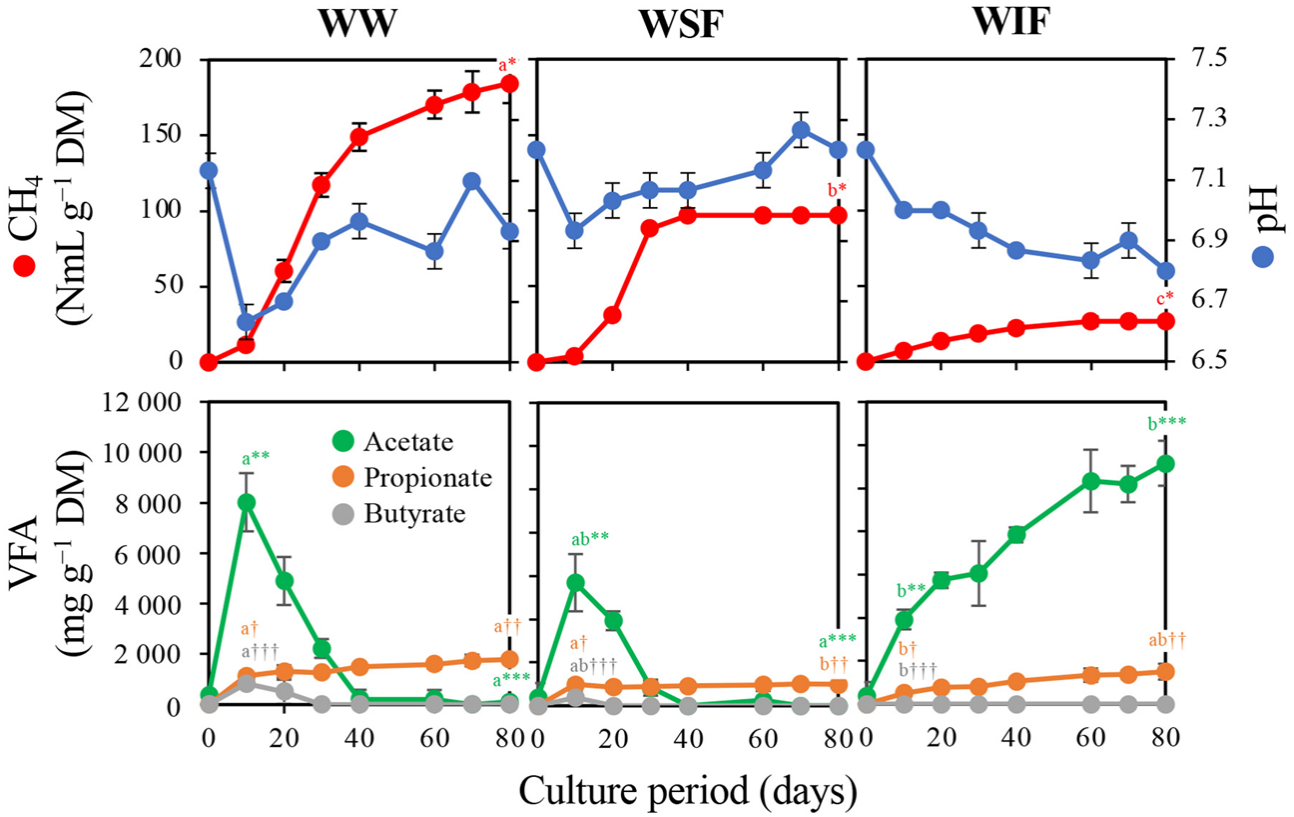
Cumulative methane production, pH changes (upper row) and VFA concentration (lower row) in the batch cultures with WW, WSF and WIF. The 80-day culture suspension of the fourth subculture with WW was used as an inoculum. Experiments were performed in triplicate and error bars represent standard deviations. The same superscripted letters indicate no significant difference (*P* < 0.05) in comparison with values with the same number of asterisks or obelisks.

Fig. 1 shows the changes in methane production and pH in batch culture with WW, WSF and WIF. The cumulative methane productions per g DM of feedstock were 184.5 NmL with WW, 96.9 NmL with WSF and 26.5 NmL with WIF.

The culture with WW showed the highest methane production. By contrast, little methane production (0.48 NmL g^−1^ DM) in the absence of weed (control) indicated that methane production in the batch culture experiment depended on the feedstock. The total methane production of culture with WSF and WIF were both significantly decreased compared with that of the culture with WW (methane production on day 80 in Fig. 1). It was suggested that the metabolites produced in the microbial degradation of WSF and WIF were mutually required for increasing the methane production because the sum of the methane production amounts of the cultures with WSF and WIF were not equal to the amount of the culture with WW. The changes in pH were similar between the cultures with WW and WSF, decreasing to 6.9 and 6.6 on day 10 and then increasing to 7.1 and 7.2, respectively. Alternatively, the pH of the culture with WIF showed a continuous decrease and finally reached 6.8 on day 80.

By analysis of VFA in cultures, acetate, propionate and butyrate were detected. In cultures with WW and WSF, acetate reached maximum concentrations on day 10 (8019.7 and 5686.9 mg L^−1^ DM, respectively), then gradually decreased and were at undetectable levels after day 40. By contrast, in culture with WIF, acetate was continuously accumulated to reach 11 157.5 mg L^−1^ DM on day 80, which was significantly higher than on day 80 in the cultures with WW or WSF (Fig. 1). From these results, it was considered that acetogenesis and methanogenesis almost proceeded, but the substrates for methanogenesis (acetate, hydrogen and carbon dioxide) were insufficient in the culture with WSF compared with that in the culture with WW. Conversely, we assumed that the microorganisms involved in conversion of acetate to hydrogen and carbon dioxide, including acetotrophic methanogens, largely decreased in the culture with WIF. To verify this assumption, we investigated the dynamics of microbial populations in the culture with WW, WSF and WIF.

### Microbial population dynamics in the culture with WW, WSF and WIF

The amplicon sequence analysis of 16S rRNA genes yielded between 159 644 and 648 130 quality-checked reads per sample obtained from the batch cultures with WW, WSF and WIF. To compare community richness and evenness, we listed the Shannon and Simpson indices in chronologically sampled cultures (Suppl. Table 1). The microbial community in the culture with WIF indicated slightly small indices (3.015 and 3.008 of Shannon index, 0.636 and 0.625 of Simpson index on days 40 and 60, respectively), whereas that in the culture with WW indicated stable values of approximately 3.5 and 0.8 of Shannon and Simpsons indices. It was noteworthy that both indices of the community in the culture with WSF drastically decreased on days 60 and 70, indicating that the richness and evenness in this community decreased. Principal component analysis and clustering of the taxonomic compositions at the genus level were also performed to visualize the differences in community structures (Suppl. Fig. 1). The plots of the communities in the same culture had a propensity to converge, except for those on day 10, and the clustering analysis clearly indicated that the taxonomical compositions of microbial communities changed with the added feedstocks (WW, WSF and WIF).

**Figure 2.**
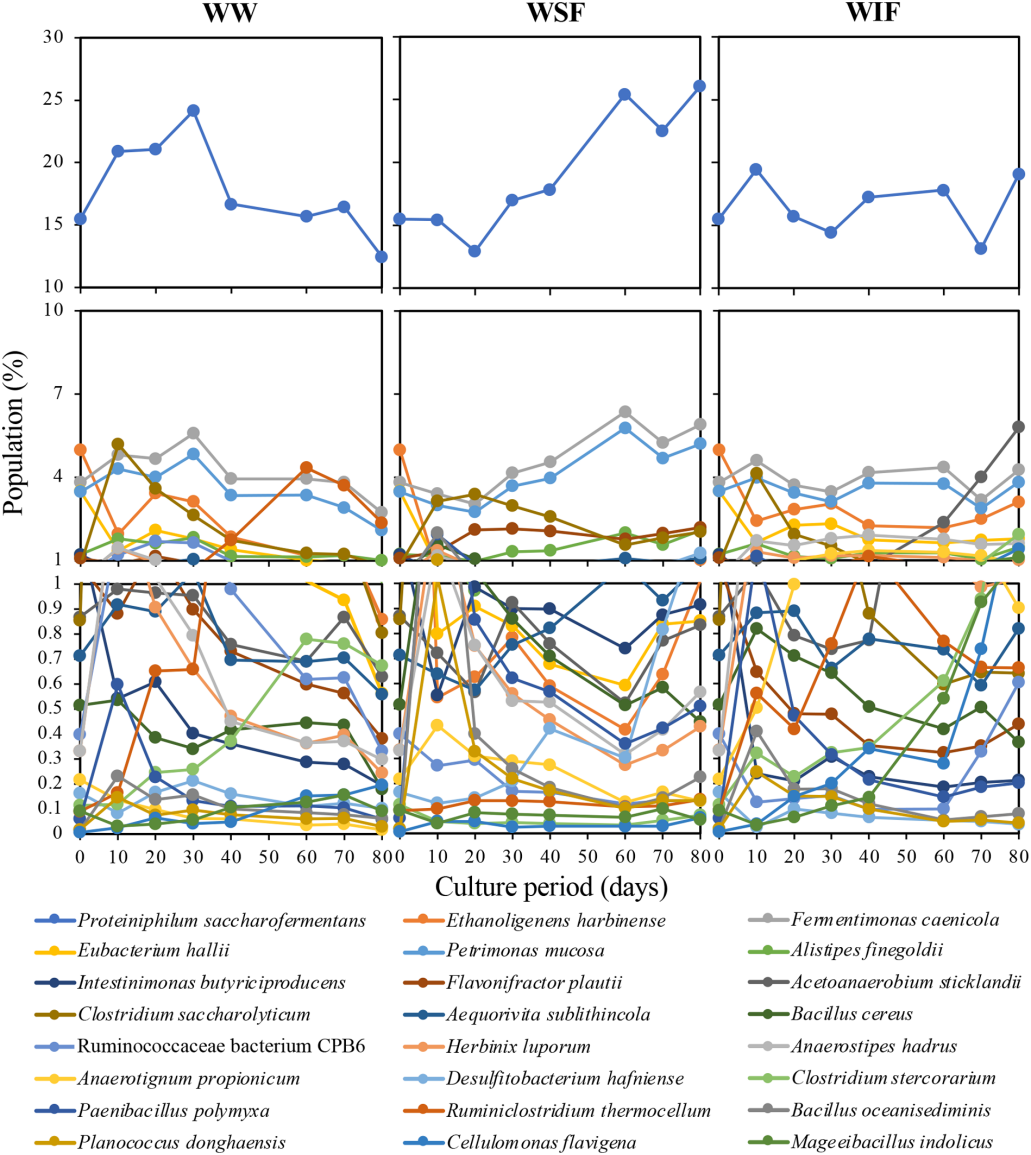
Changes in eubacterial population at the species level in the cultures shown in Fig. 1. Populations of the species that once exceeded 1% are indicated in every culture.

**Figure 3.**
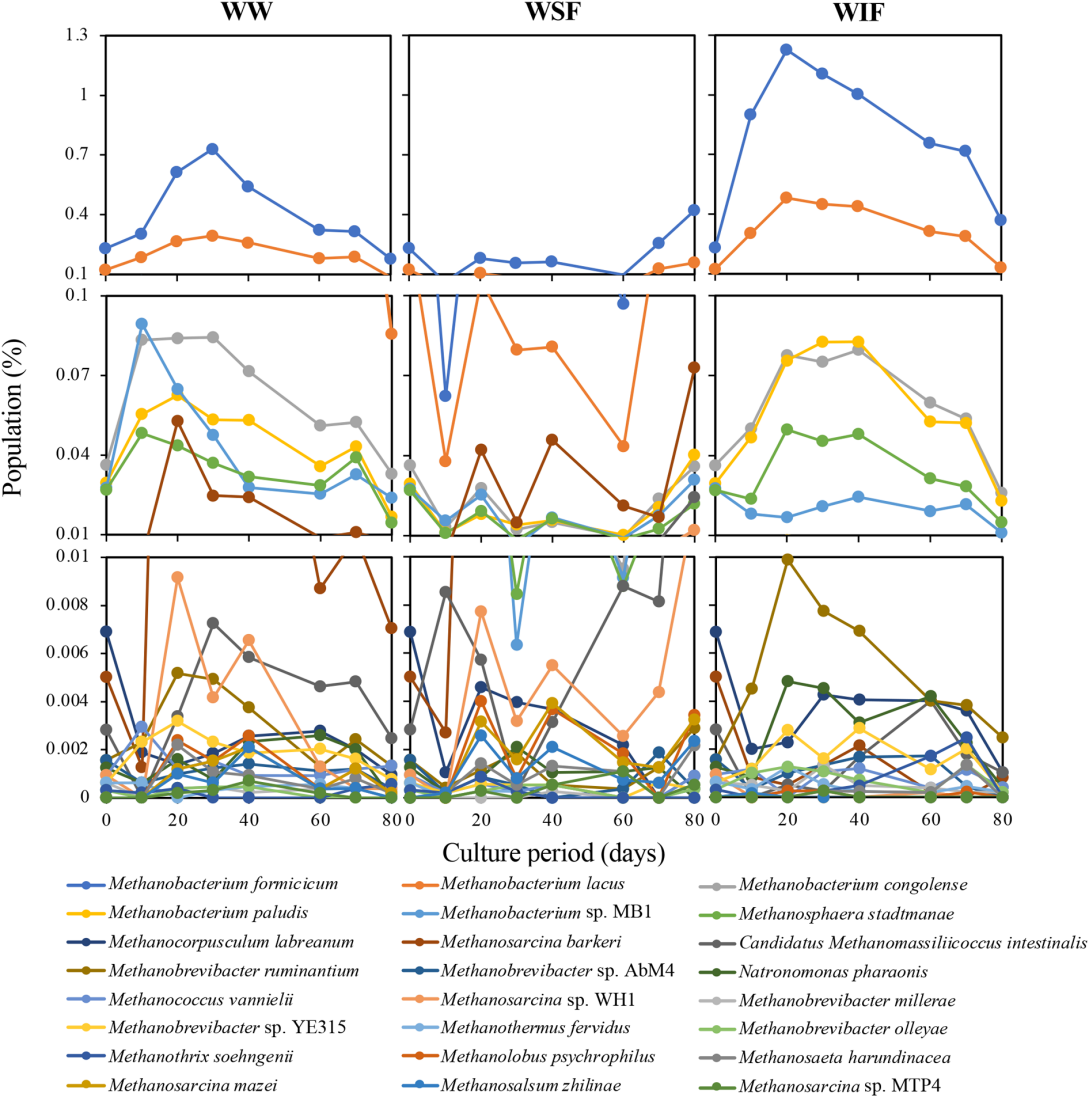
Changes in archaeal population at the species level in the cultures shown in Fig. 1. Populations of the species that once exceeded 0.001% in all-over culture are indicated in every culture.

To visually depict the shift of members in the microbial community, we illustrated the ‘growth curve-like’ figures at the species level based on the assignment data from the WIMP workflow. Figs 2 and 3 show the changes in species that exceeded 1% for eubacteria and 0.001% for archaea in total population during the culture, respectively. The dominant species conformed to those in the microbial communities of our previous study (Matsuda and Ohtsuki 2016), suggesting that the culture acclimation reappeared.

The most dominant species *Proteiniphilum saccharofermentans* consistently comprised >10% of the population, 12.5–24.1% with WW, 15.5–26.1% with WSF and 13.2–19.4% with WIF (Fig. 2). *Proteiniphilum saccharofermentans* was isolated from the biogas reactor and was reported to have degradative activity for various sugars such as cellobiose and xylan and to produce acetate, propionate and isovalerate (Hahnke *et al*. 2016). The metabolic pathways have also been investigated and involvement in substrate hydrolysis and acid formation in the biogas production process has been suggested (Tomazetto *et al*. 2018).

Comparing the changes among cultures with WW, WSF and WIF, the population ratio of *Ethanoligenens harbinense* and *Eubacterium hallii* in the culture with WSF decreased to <1% (Fig. 2). *Ethanoligenens harbinense* and *Eu. hallii* have been reported to produce hydrogen (Duncan, Louis and Flint 2004; Xu *et al*. 2008) and a decrease in these species might be associated with the repression of hydrogenotrophic methanogenesis. The population of *Cellulomonas flavigena* gradually increased in the culture with WIF and finally reached 1.4%. The population of *Ce. flavigena* was <0.2% and <0.1% in the cultures with WW and WSF, respectively. *Cellulomonas flavigena* could assimilate cellulose (Ponce-Noyola and Torre 1995), and an increase in the population of *Ce. flavigena* observed in the culture with WIF could be attributed to an increased ratio of crystalline cellulose in WIF. The population of *Acetoanaerobium sticklandii* increased significantly only in the culture with WIF (up to 5.8%), whereas the population ranged from 0.1 to 0.9% in the culture with WW or WSF. *Acetoanaerobium sticklandii* is known to produce acetate (Sangavai and Chellapandi 2019) and was inferred to contribute to acetate accumulation in the culture with WIF.

*Methanobacterium formicicum* can produce methane from hydrogen and carbon dioxide (Chellapandi *et al*. 2018). *Methanobacterium formicicum* showed the highest population in all cultures, suggesting it to be the main species in methanogenesis. The population of several archaeal species peaked similarly at around day 20 in cultures with WW or WIF compared with the constantly low population of the species in the culture with WSF. Remarkably, it was considered that a decrease in the population of acetate-assimilating methanogen *Methanosarcina barkeri* was associated with acetate accumulation in the culture with WIF because the population of this species was apparently smaller (<0.001–0.005%) than the population in the culture with WW or WSF (0.005–0.073%).

### Re-mixed culture of subcultures with WSF and WIF

**Figure 4.**
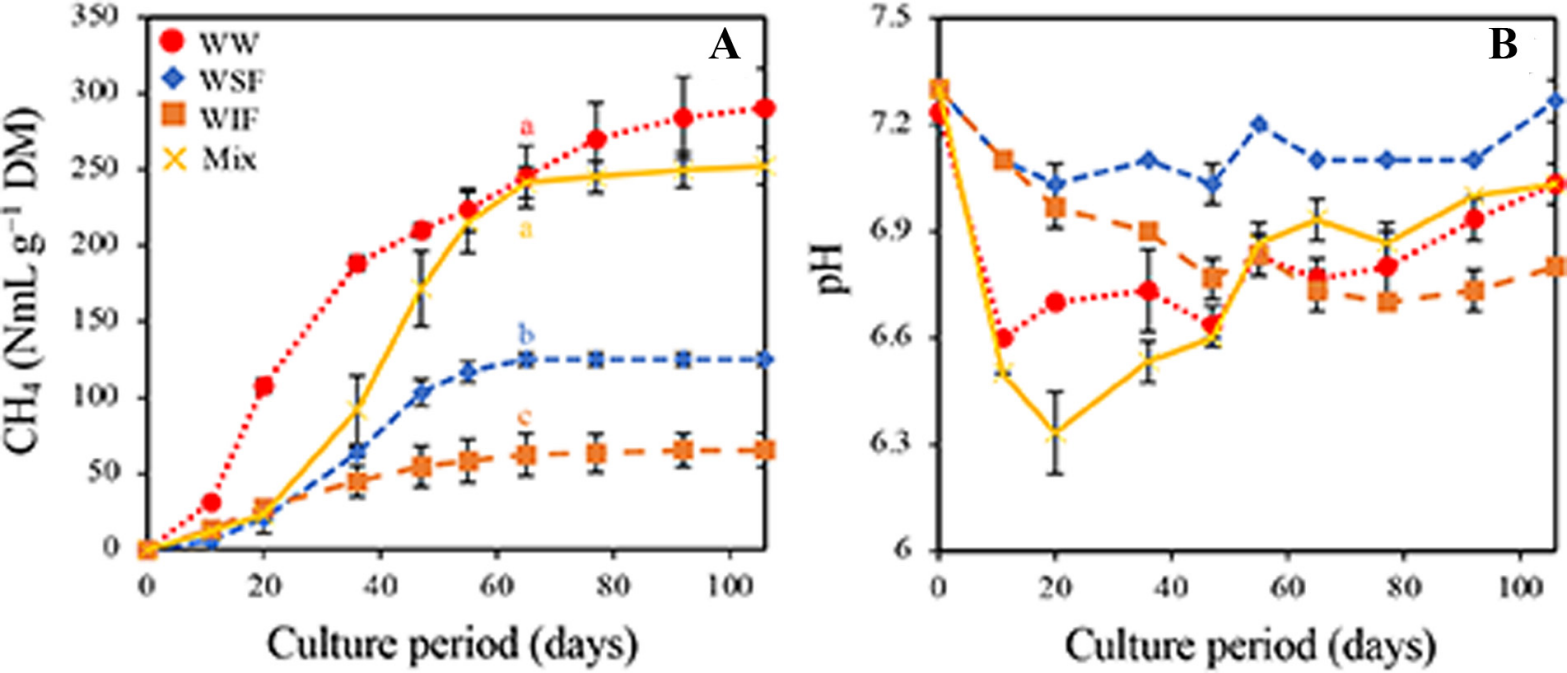
Comparison of cumulative methane production (**A**) and pH changes (**B**) among subcultures and re-mixed culture. WW, eighth subculture with WW; WSF and WIF, third subcultures with WSF and WIF, respectively; Mix, re-mixed culture with WW. Experiments were performed in triplicate and error bars represent standard deviations. The same letters in methane production on day 65 indicate no significant difference (*P* < 0.05).

Microbial communities acclimated to WSF or WIF were mixed as a source of inoculum and the recovery of methane-producing ability was examined using WW as a substrate. The cumulative methane production reached 252.4 compared with 290.5 NmL g^−1^ DM of the production of the culture with WW by inoculation of the subcultures with WW (Fig. 4). The cumulative methane productions of the subcultures were 125.1 NmL g^−1^ DM with WSF and 65.4 NmL g^−1^ DM with WIF. Notably, the methane production in the re-mixed culture slowly increased compared with that in the subculture with WW; however, the production was similar with that in the subculture with WW on day 65 (245.2 NmL g^−1^ DM in subculture and 241.3 NmL g^−1^ DM in re-mixed culture). The pH in the re-mixed culture decreased to 6.3 on day 20, whereas the pH in the subculture with WW decreased to 6.6 on day 10, in which the difference in pH change might cause a delay in methane production. These results strongly supported the importance of interactions between the microorganisms that assimilated WSF and WIF. Further metatranscriptomics and metabolomics analyses will be conducted to determine the exchange of metabolites in the microbial community.

To investigate the changes in the microbial population in the re-mix culture, 16S rRNA gene amplicon analysis was also carried out using a nanopore sequencer. The total population (100%), comprising 177 864–316 670 qualified reads, including all assigned and unassigned sequences, was analyzed. The Shannon indices were 2.153–2.562 and 2.306–2.600 and the Simpson indices were 0.475–0.536 and 0.485–0.570 for the subculture with WW and the re-mixed culture, respectively. These index values suggested that the richness and evenness were both decreased (acclimation of the community had progressed) in comparison with the initial subcultures. In addition, the illustration of the results from principal component analysis and clustering showed that the inoculum from the culture with WSF to the re-mixed culture apparently had a different microbial community structure; however, the plots of the re-mixed culture were close to those of the subculture with WW (Suppl. Fig. 2). Because several taxonomic components from both cultures were clustered (e.g. WW8th_Day11 and Mix_Day20 in Suppl. Fig. 2), it was considered that the colocalization of microbial community in the subculture with WW had been reconstructed in the re-mixed culture.

Supplementary Figs 3 and 4 show the changes in population ratio of the subculture with WW and re-mixed culture. In both cultures, although the dominant eubacterial species were *Pr. saccharofermentans*, *Fermentimonas caenicola* and *Petrimonas mucosa*, the dominant archaeal species were *Methanobacterium formicicum* and *Methanobacterium lacus*. As the culture with WW in this experiment was the eighth subculture, other minor eubacterial species observed in the fifth subculture (Figs 2 and 3) were present but at negligible concentrations (<0.001%). Therefore, the species in the culture were more acclimated with subculturing, but the eliminated minor species were considered to be unassociated with methane production. Of particular interest, *Pr. saccharofermentans*, *F. caenicola* and *Pe. mucosa* were reported to be isolated from the same fermenter and were considered to collaborate for acidogenesis (Hahnke *et al*. 2016). Eubacterial population changes of <3% level differed despite the production of similar amounts of methane in the re-mixed culture compared with the subculture with WW, and most archaeal species tended to decrease during culture. The populations of *Ruminiclostridium thermocellum* (0.14–1.75% in the subculture and 0.03–0.14% in the re-mixed culture), *Cl. saccharolyticum* (1.21–2.86% in the subculture and 0.60–2.09% in the re-mixed culture), *Ruminococcaceae* bacterium CPB6 (0.36–0.65% in the subculture and 0.14–2.43% in the re-mixed culture), *Intestinimonas butyriciproducens* (0.26–0.51% in the subculture and 0.24–1.49% in the re-mixed culture), *Et. harbinense* (1.09–1.21% in the subculture and 1.97–3.21% in the re-mixed culture) and *Eu. hallii* (0.99–1.14% in the subculture and 1.69–2.46% in the re-mixed culture) identified under 3% level in the eubacterial species fluctuated (Suppl. Fig. 3). Because the contribution of these minor species to methane production remains unknown, experiments including the isolation of these species and reconstruction of the methane-producing community with WW are currently underway.

In the archaeal species, *Methanosarcina barkeri* was found with <0.001–0.002% and 0.002–0.006% of population in the re-mixed culture and subculture with WW, respectively, except on day 0 (Suppl. Fig. 4). We showed that *Methanosarcina barkeri* possibly assimilated acetate in the culture with WIF; however, this species had little possibility to contribute to methane production. Combining the results from the culture with WSF or WIF and the re-mixed culture strongly suggested that converting acetate to hydrogen and carbon dioxide was almost processed by the eubacterial species and methane was mainly produced by the hydrogenotrophic *Methanobacterium* species.

## CONCLUSION

Methane production in culture with WSF or WIF and the re-mixed culture were compared with its production with WW and population dynamics were characterized by nanopore sequencing analysis. In the methane production, the drastic decrease in culture with WSF or WIF and complete recovery in the re-mixed culture clearly showed the importance of the coexistence of the community members mainly assimilating WSF and WIF. In addition to an effort to isolate the dominant species in the microbial community, we are planning to reconstitute a methane-producing community with type strains of the major assigned species. The continuation of our research will provide useful insights into the effective use of weed (not only Gyogi-shiba) as a sole feedstock for methane production.

## Supplementary Material

fnab015_Supplemental_Files
