# Supplementary material for: Effective methane production from the Japanese weed Gyougi-shiba (Cynodon dactylon) is accomplished by colocalization of microbial communities that assimilate water-soluble and -insoluble fractions
Source: FEMS Microbiol Lett. 2021 Feb 15;368(4):fnab015. doi: 10.1093/femsle/fnab015 (PMC7939696; doi:10.1093/femsle/fnab015)
Supplement: fnab015_Supplemental_Files [file fnab015_supplemental_files.zip › SupplementalyTable_Matsuda&Ohtsuki.docx]

**Supplementary Table 1.** The Shannon and Simpson indices of microbial communities at the genus level

| Culture  with: | Shannon index | | | | | | |  | Simpson index | | | | | | |  |
| --- | --- | --- | --- | --- | --- | --- | --- | --- | --- | --- | --- | --- | --- | --- | --- | --- |
|  | 10 | 20 | 30 | 40 | 60 | 70 | 80 |  | 10 | 20 | 30 | 40 | 60 | 70 | 80 | (day) |
| WW | 3.662 | 3.743 | 3.726 | 3.555 | 3.776 | 3.516 | 3.477 |  | 0.838 | 0.830 | 0.831 | 0.713 | 0.782 | 0.749 | 0.817 |  |
| WSF | 3.415 | 3.414 | 3.238 | 3.244 | 1.918 | 2.192 | 3.412 |  | 0.835 | 0.726 | 0.683 | 0.665 | 0.396 | 0.493 | 0.764 |  |
| WIF | 3.641 | 3.482 | 3.589 | 3.015 | 3.008 | 3.563 | 3.076 |  | 0.848 | 0.716 | 0.752 | 0.636 | 0.625 | 0.754 | 0.777 |  |

The Shannon and Simpson indices of microbial community on day 0 were 3.586 and 0.734, respectively.
