# Supplementary material for: Effective methane production from the Japanese weed Gyougi-shiba (Cynodon dactylon) is accomplished by colocalization of microbial communities that assimilate water-soluble and -insoluble fractions
Source: FEMS Microbiol Lett. 2021 Feb 15;368(4):fnab015. doi: 10.1093/femsle/fnab015 (PMC7939696; doi:10.1093/femsle/fnab015)
Supplement: fnab015_Supplemental_Files [file fnab015_supplemental_files.zip › SupplFigureLegends_Matsuda&Ohtsuki.docx]

**Supplementary Figure legends**

**Supplementary Figure 1.** Principal component analysis based on the taxonomic profile of samples from the culture with WW, WSF, and WIF. Annotated numbers of OTUs were log-transformed. Unit variance scaling was applied to rows and singular value decomposition with imputation was used to calculate the principal components. Samples were also clustered using correlation distance and average linkage. Plots belonging to same clusters are indicated the same colors.

**Supplementary Figure 2.** Principal component analysis based on the taxonomic profile of samples from the 8th subculture with WW and re-mixed culture with WW. Annotated numbers of OTUs were log-transformed. Unit variance scaling was applied to rows and singular value decomposition with imputation was used to calculate the principal components. Samples were also clustered using correlation distance and average linkage. Plots belonging to same clusters are indicated by same colors.

**Supplementary Figure 3.** Changes in eubacterial population at the species level in the cultures shown in Fig. 4. WW - 8th subculture with WW; Mix - re-mixed culture with WW. Population of the species that once exceeded 1% in all-over culture is indicated in every culture.

**Supplementary Figure 4.** Changes in archaeal population at the species level in the cultures shown in Fig. 4. WW - 8th subculture with WW; Mix - re-mixed culture with WW. Population of the species that once exceeded 0.001% in all-over culture is indicated in every culture.
