# Supplementary figures and images for: Effective methane production from the Japanese weed Gyougi-shiba (Cynodon dactylon) is accomplished by colocalization of microbial communities that assimilate water-soluble and -insoluble fractions
Source: FEMS Microbiol Lett. 2021 Feb 15;368(4):fnab015. doi: 10.1093/femsle/fnab015 (PMC7939696; doi:10.1093/femsle/fnab015)

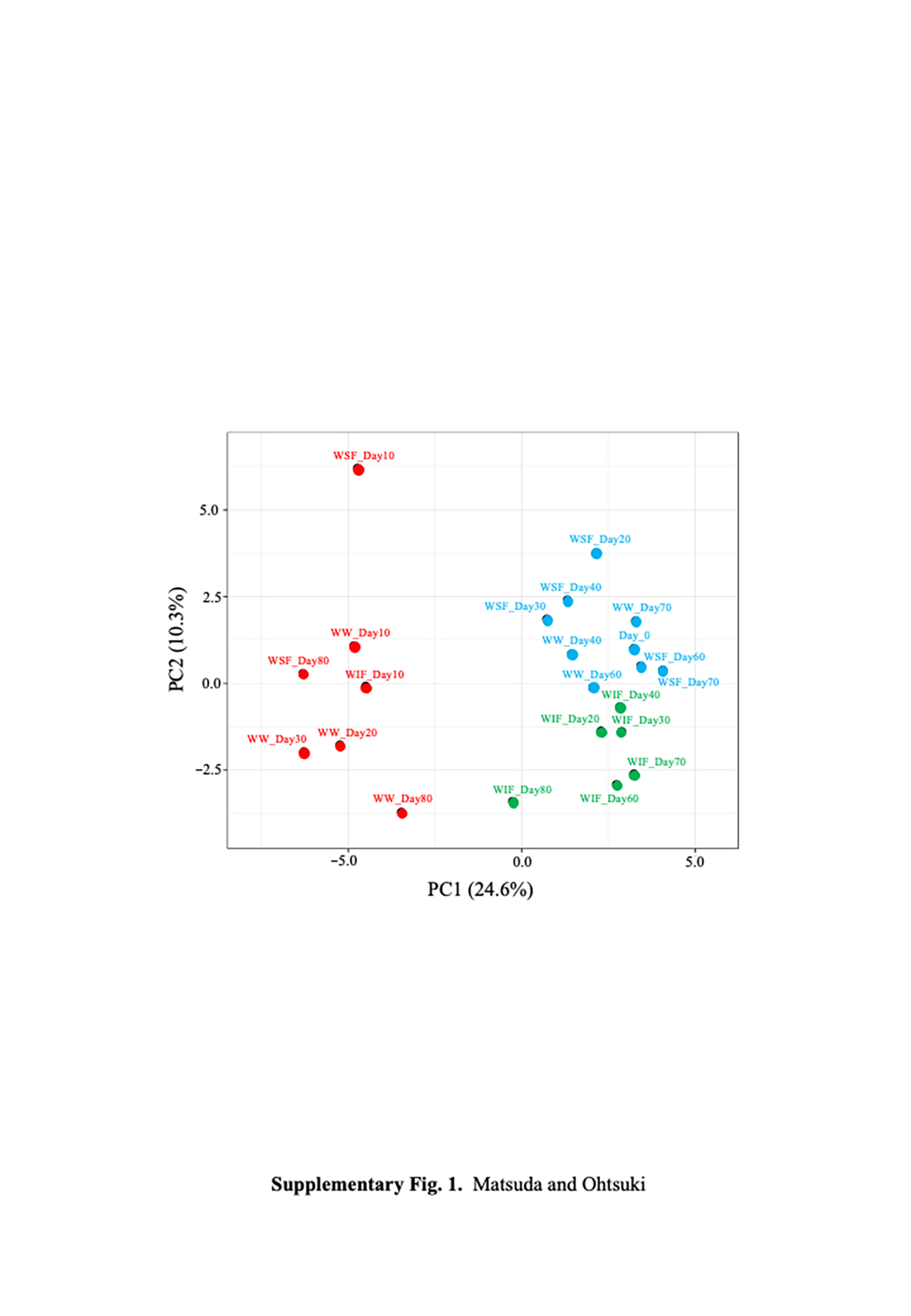

Supplement: fnab015_Supplemental_Files [file fnab015_supplemental_files.zip › SupplFigure1_Matsuda&Ohtsuki.tiff]

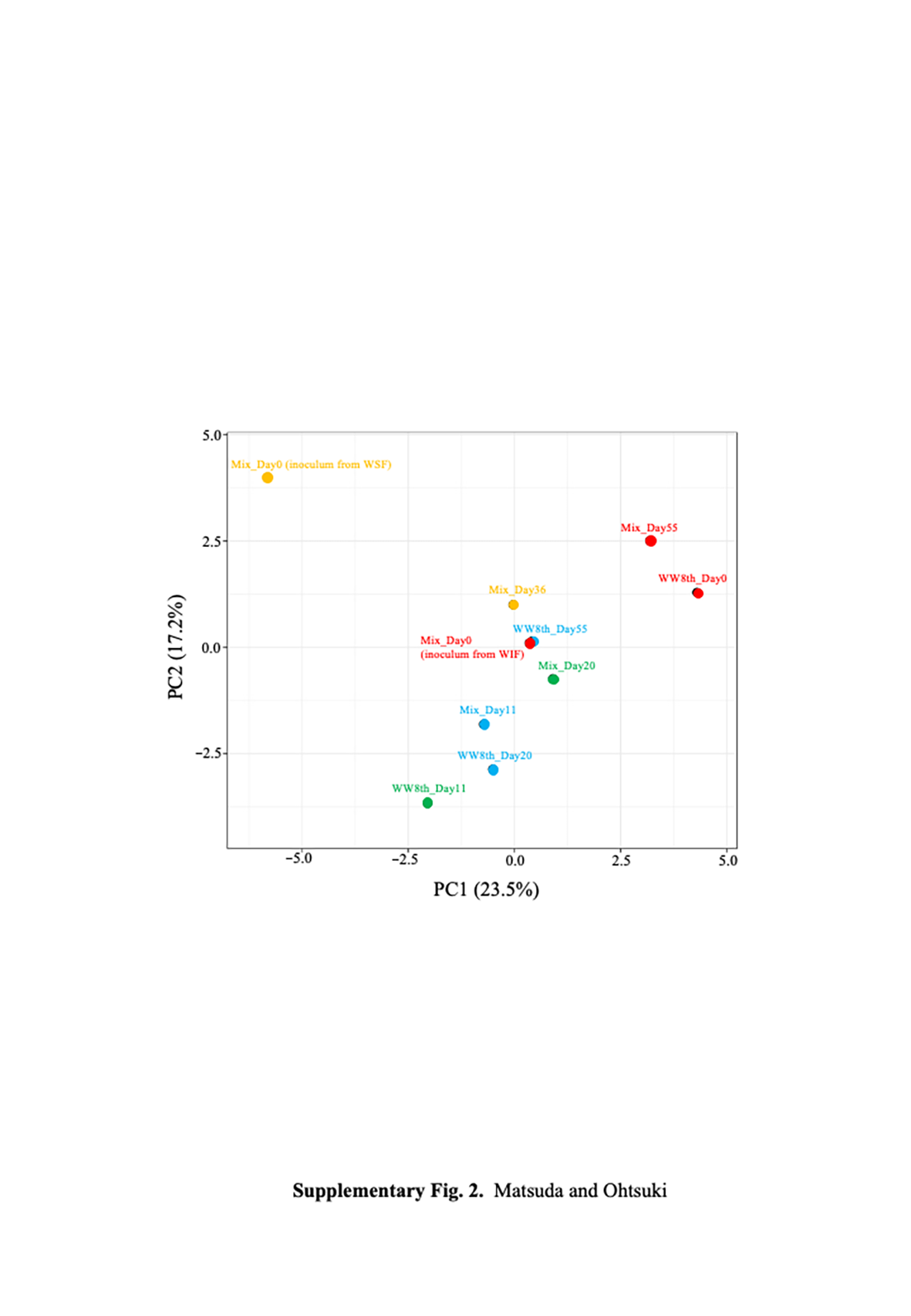

Supplement: fnab015_Supplemental_Files [file fnab015_supplemental_files.zip › SupplFigure2_Matsuda&Ohtsuki.tiff]

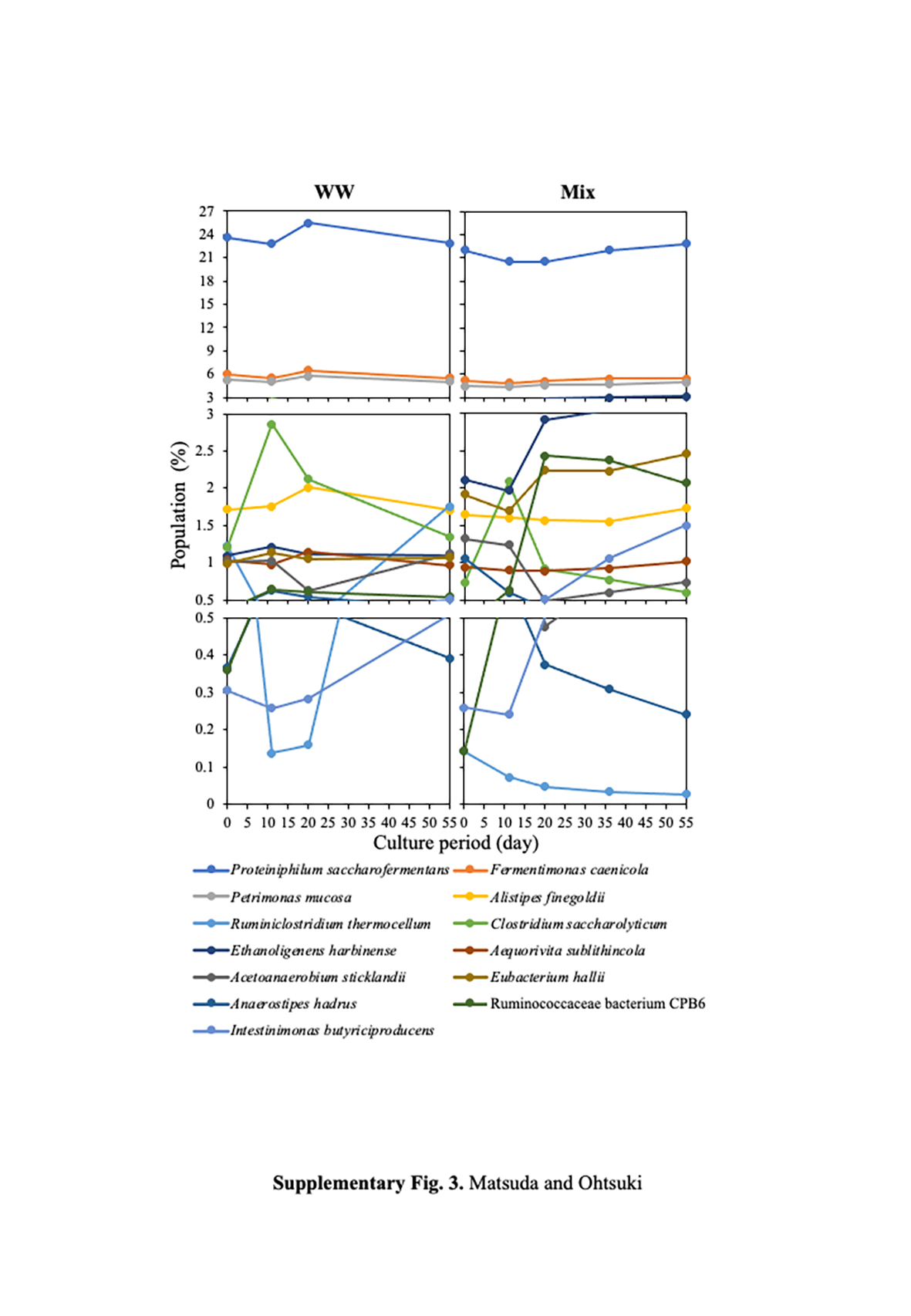

Supplement: fnab015_Supplemental_Files [file fnab015_supplemental_files.zip › SupplFigure3_Matsuda&Ohtsuki.tiff]

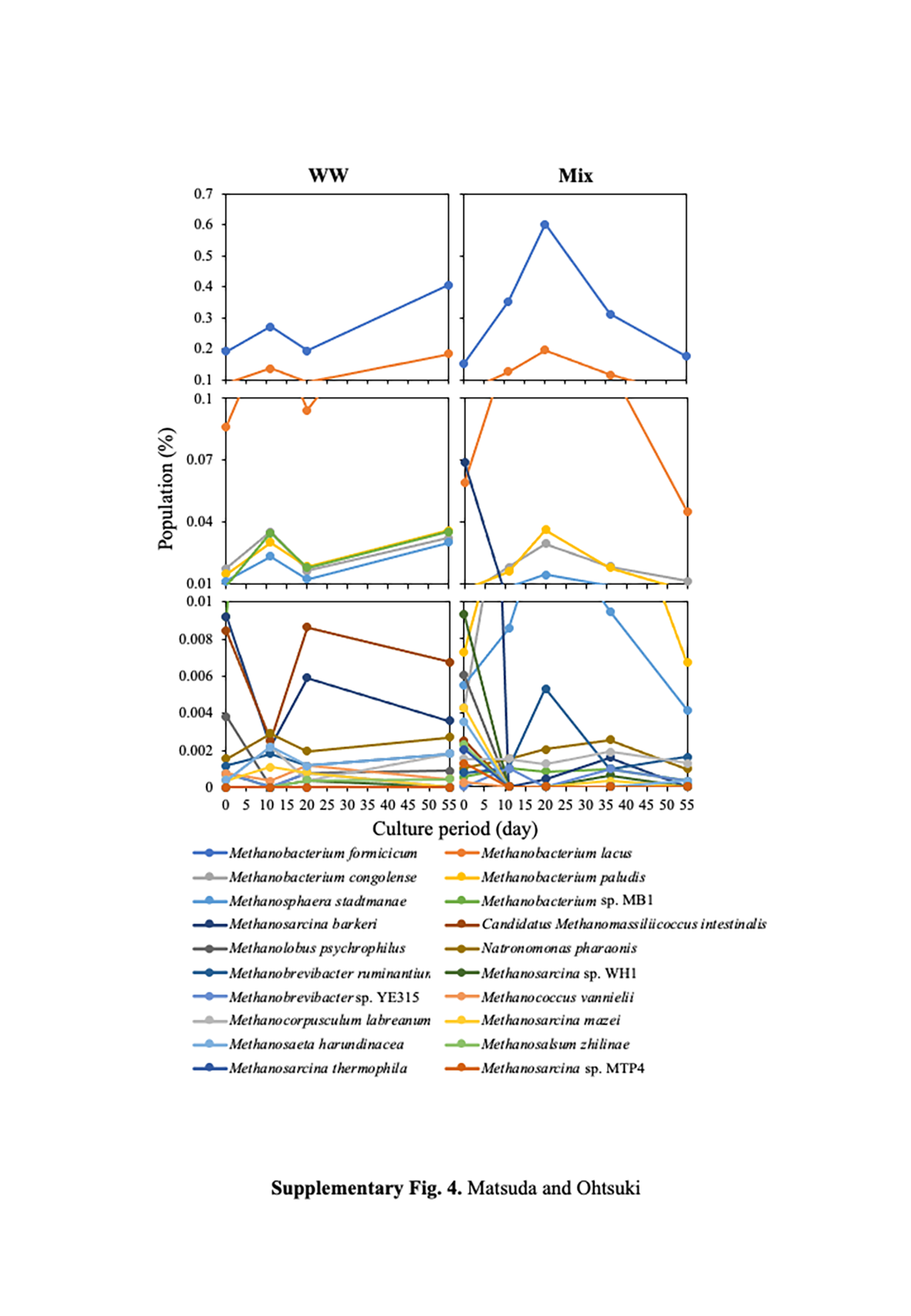

Supplement: fnab015_Supplemental_Files [file fnab015_supplemental_files.zip › SupplFigure4_Matsuda&Ohtsuki.tiff]
